# Women with depression in pregnancy or a history of depression have decreased quality of mentalization in the speech to their infants

**DOI:** 10.1111/acps.13624

**Published:** 2023-11-06

**Authors:** Lavinia Rebecchini, Rebecca H. Bind, Beatrice Allegri, Arianna Zamparelli, Alessandra Biaggi, Katie Hazelgrove, Sarah Osborne, Susan Conroy, Susan Pawlby, Vaheshta Sethna, Carmine M. Pariante

**Affiliations:** 1 Department of Psychological Medicine, Institute of Psychiatry, Psychology and Neuroscience (IoPPN), King’s College London, London, UK; 2 Department of Forensic & Neurodevelopmental Sciences, Sackler Institute for Translational Neurodevelopment, Institute of Psychiatry, Psychology & Neuroscience (IoPPN), King’s College London, London, UK

**Keywords:** cognitive biases, depressive disorders, maternal speech, mentalization, mother-infant interaction

## Abstract

**Background:**

Our study aims to understand whether depression, either in pregnancy or lifetime, affects cognitive biases (comprising the attentional focus and affective state) and mentalizing features (ability to understand children’s internal mental states, thereby detecting and comprehending their behavior and intention), in maternal speech during mother-infant interaction in the first postnatal year.

**Methods:**

We recruited 115 pregnant women (44 healthy, 46 with major depressive disorder [MDD] in pregnancy, and 25 with a history of MDD but healthy pregnancy) at 25 weeks’ gestation. Three-minute videos were recorded at 8 weeks and 12 months postnatally for each dyad. Maternal speech was transcribed verbatim and coded for cognitive biases and mentalizing comments using the Parental Cognitive Attributions and Mentalization Scale (PCAMs).

**Results:**

Women suffering from antenatal depression showed a decreased proportion of mentalizing comments compared with healthy women, at both 8 weeks (0.03 ± 0.01 vs. 0.07 ± 0.01, *P* = 0.002) and 12 months (0.02 ± 0.01 vs. 0.04 ± 0.01, *P* = 0.043). Moreover, compared with healthy women, both those with antenatal depression and those with a history of depression showed decreased positive affection in speech (0.13 ± 0.01 vs. 0.07 ± 0.01 and 0.08 ± 0.02, respectively *P* = 0.003 and *P* = 0.043), and made significantly fewer comments focused on their infants’ experience at 8 weeks (0.67 ± 0.03 vs. 0.53 ± 0.04 and 0.49 ± 0.05, respectively *P* = 0.015 and *P* = 0.005). In linear regression models women’s socioeconomic difficulties and anxiety in pregnancy contribute to these associations, while postnatal depression did not.

**Conclusions:**

Both antenatal depression and a lifetime history of depression are associated with a decreased quality of women’s speech to their infants, as shown by less focus on their infant’s experience, decreased positive affection, and less able to mentalize. Examining maternal speech to their infants in the early postnatal months may be particularly relevant to identify women who could benefit from strategies addressing these aspects of the interactive behavior and thus improve infant outcome in the context of depression.

## 1 Introduction

Depression in pregnancy is associated with offspring adverse outcomes, impacting child language development and cognitive functioning, and increasing the risk of socio-emotional difficulties.^1,2^ One possible pathway by which maternal depression leads to adverse child outcomes is through its effect on maternal communication during mother-infant interactions. Our previous study in the PRAM-D cohort^3^ reported that both antenatal depression (AND) and a lifetime history of depression prior to pregnancy are associated with a decreased quality of mother-infant interaction across the postnatal period, as captured by the Crittenden Child-Adult Relationship Experimental-Index (CARE-Index)—an observational measure of the mother−child relationship.^4^ However, analysis of maternal speech to infants during mother-infant interaction may also be relevant to identifying aspects of interactive behavior that affect child’s development. In fact, studies have shown that maternal speech contributes to the child’s emotional and cognitive development,^5,6^ and caregiver’s mentalizing ability, hence the parents’ capacity and interest to consider the individual experience and mental state underlying the behavior s of the child, in the first year of life predicts attachment security^7,8^ and children’s theory of mind abilities at age 4 years.^9^

Two key features of maternal speech are cognitive biases,^10^ comprising the attentional focus and affective state of the speech, and mentalization,^11^ the ability to understand children’s mental states, thereby detecting and comprehending their behavior and intentions. Both these features are influenced by parental attribution of agency,^12^ reflective functioning,^13,14^ and parental mind-mindedness (i.e., the caregiver’s tendency to view their child as an individual with their own thoughts, feelings and desires),^15^ all relevant to recognizing and interpreting mental processes and creating a “meeting of minds” between parent and child.^16^ The present study examines maternal cognitive and mentalizing features in maternal speech to their infants in the same PRAM-D cohort.

To our knowledge, studies on cognitive and mentalizing features in maternal speech have focused on mothers with postnatal depression (PND), showing that they comment less on their infants’ emotional and cognitive mental states, are less likely to acknowledge infant intentions and agency, and express more negative affect.^12,17,18^ However, little is known about whether maternal depression experienced prior to the postnatal period—that is, in pregnancy or even prior to pregnancy—may have the same impact.Thus, in the present study, we explore whether there is an effect of women’s clinical depression (either during pregnancy or in lifetime before pregnancy) on the attentional focus and affective quality of mothers’ speech (cognitive biases), and on their use of mentalizing comments, at 8 weeks and 12 months postnatal. Furthermore, we investigate whether certain clinical and sociodemographic risk factors (such as maternal childhood maltreatment, socio-economic stress, anxiety, PND, suboptimal neonatal behavior, and reduced dyadic synchrony) that are known to be associated with maternal depression^19,20^ and with maternal sensitivity^3,21^ are also associated with mentalization in the current sample.^22,23^

## 2 Method

### 2.1 Study design

**Figure 1:**
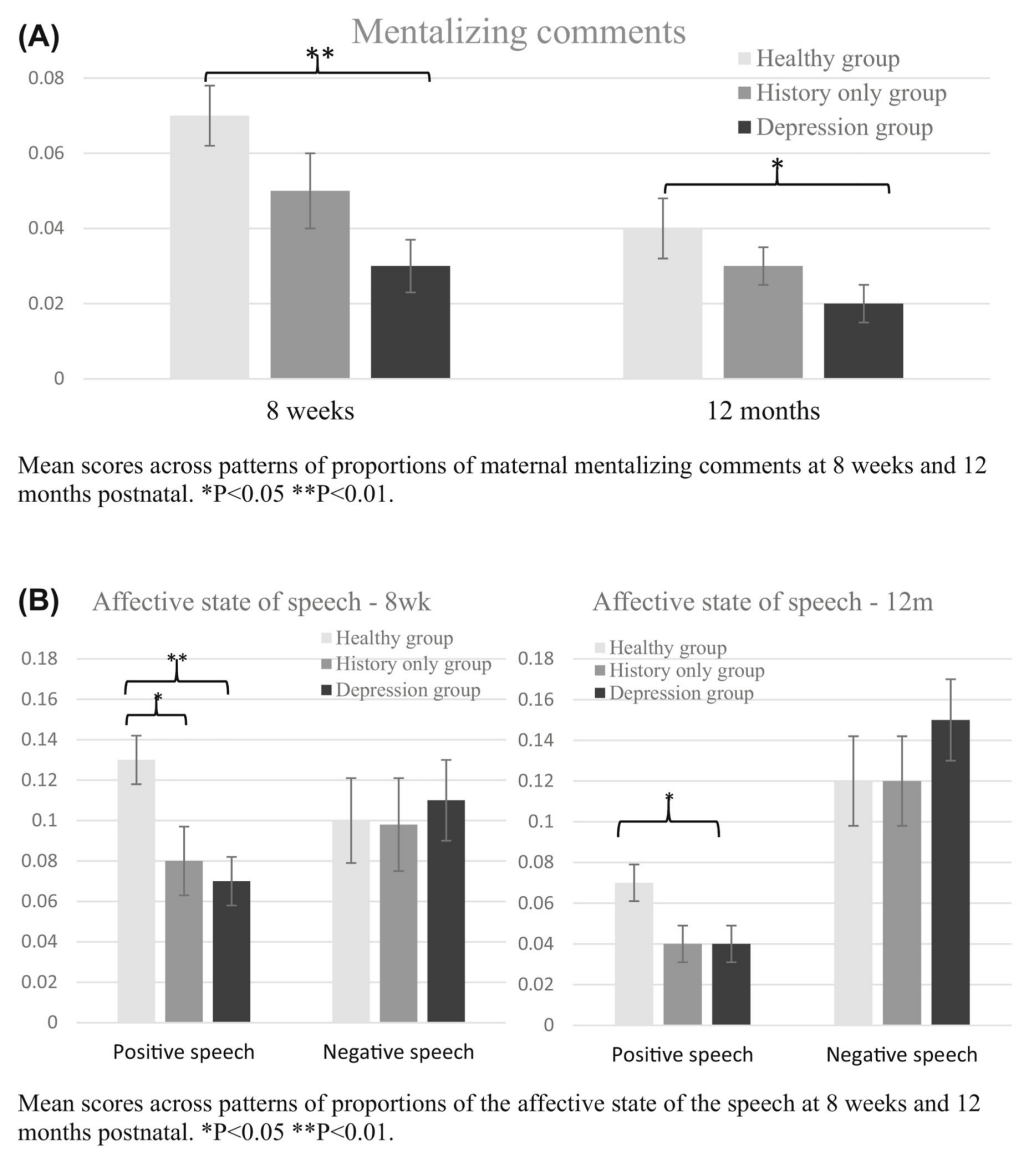
Mean scores across proportions of maternal speech on the Parental Cognitive Attribution and Mentalization Scale at 8 weeks and 12 months postnatal. *P < 0.05, **P < 0.01.

The Psychiatry Research and Motherhood − Depression study (PRAM-D)^24^ is a prospective longitudinal study of women in pregnancy and postpartum, and their off-spring. As this sample has been extensively described before,^3,24,25^ details of the general participants’ features and assessment procedures are described in the Supplementary Material S1 (Study design and Figure 1), while only the most relevant points are summarized here.

### 2.2 Participants and procedure

The sample of the present study includes 115 pregnant women: 44 ‘healthy’, with no present or past mental health diagnosis, attending their routine antenatal ultrasound scan at King’s College Hospital [healthy group]; 46 diagnosed with major depressive disorder (MDD) in pregnancy but no history of depression prior to the current pregnancy, referred to the Maudsley Perinatal Psychiatry Services [depression group]; and 25 ‘history-only’, with a history of MDD but no current depression, recruited from either their regular antenatal scan or the psychiatric clinical service (where they were referred for assessment only, because of the historical vulnerability) [history-only group]. All women and their offspring were assessed from 25 weeks’ gestation (baseline) until 1 year postnatal. Of the 115 women assessed at baseline for sociodemographic and clinical information, 114 dyads were seen at 6 days postnatal to assess neonatal behavior, 101 dyads were seen at 8 weeks postnatal, and 98 dyads were seen at 12 months postnatal, to assess sociodemographic and clinical vari-ables, as well as the mother−infant interaction. Procedures were approved by the King’s College Hospital Research Ethics committee, REC 07/Q0703/48, and comply with the Helsinki Declaration of 1975, as revised in 2008. All participants provided written informed consent.

### 2.3 Measures

#### 2.3.1 Sociodemographic and clinical measures

As previous studies have shown, sociodemographic/economic (SES) variables were very highly correlated with each other, and thus a composite SES score was created to express cumulative risk.^3,26^ Variables included maternal age, ethnicity (white versus ethnic minority), marital status (married or cohabiting versus single with or without partner), occupation (employed or student versus non-employed or full-time mother) and qualification level (GCSE or lower versus A-level or higher), all assessed at baseline. A score of 0 represented the mean status across the sample, a negative score represented more risk factors for adversity and a positive score represented more protective factors. These particular SES variables were chosen as young age, belonging to an ethnic minority group, being single, being unemployed and having lower education qualifications have all been previously associated with maternal depression.^27^

Measures of maternal history of sexual, physical, emotional abuse, and neglect were obtained using the Childhood Experience of Care and Abuse Questionnaire (CECA-Q).^28^

Past and current Axis I diagnosis, including MDD, were assessed at baseline using the *Structured Clinical Interview for DSM-IV Disorders* (*SCID-I*). Participants were readministered the SCID-I at 8 weeks and 12 months postnatal, to assess for episodes of PND since the previous assessment. The Beck Depression Inventory (BDI) and State−Trait Anxiety Inventory (STAI) were self-administered at baseline, week 8 and month 12 to assess symptoms of current depression and anxiety. All interviews were conducted by trained graduate psychologists or psychiatrists, and a consensus was reached on diagnoses.

#### 2.3.2 Mother and infant behaviors

Neonatal behavior was evaluated at 6 days postnatal using the Neonatal Behavioral Assessment Scale (NBAS),^29^ describing the neonates’ responses to the new extrauterine environment; in this study, we only include the “orientation” cluster, as it assesses the infant’s ability to attend to visual and auditory stimuli, their overall quality of alertness and their social-interactive abilities, and it is associated with dyadic interaction.^3,30^

Quality of the interaction and infants’ behavior were assessed using the CARE-Index,^4^ at both 8 weeks and 12 months, as previously described.^3^ The “dyadic synchrony” cluster was utilized as a global indication of how attuned the dyad is and how well mothers and infants are interacting, in addition to “infant difficultness”, presenting as negative connectedness to the mother and over-arousal. Infant difficultness was included to account for the role of infant’s behavior on maternal affect in speech, based on a previous study, that highlighted that infant-directed negativity in a depressed parent’s speech was not associated with infant’s fretfulness.^31^

#### 2.3.3 Maternal speech

Three-minute videos of the mother-infant interaction were filmed at 8 weeks and 12 months postnatal in the families’ homes. Mothers were instructed to play with their babies as they would normally do. Maternal speech was transcribed verbatim from videotapes of the interaction. An “utterance” was defined as a group of words bound by silence, a short pause, or an intonation to signal a question mark or period at the end of the sentence or thought.^31^ Single words, sentence fragments, non-verbal sounds and songs were each counted as an utterance. In addition to transcribing maternal utterances, the context in which they were made was also recorded. Videotapes were coded by two researchers blind to maternal depression status. Individual variable scores were calculated by summing the total number of utterances, and analyses were conducted using proportions of the total number of utterances. Reliability was established using the intraclass correlation coefficient (ICC) between the two trained raters, blind to maternal mental health status, with an ICC of 0.90 for the proportion of mentalizing comments at 8 weeks, and an ICC of 0.88 at 12 months. Videos in foreign languages or with poor quality of sound (i.e., inaudible speech) were excluded (*n* = 14 at 8 weeks; *n* = 17 at 12 months: these subjects are not included in the sample description in Table 1).

Maternal speech was assessed using The Parental Cognitive Attributions and Mentalizing Scale (PCAMs) (manual available from the authors upon request),^31^ which examines parental speech during parent-infant interactions with infants up to 12 months. The scale was adapted and developed from previous work by Murray,^12,32^ and Meins.^15^ The scale rates: (I) Mentalizing comments (maternal awareness and interpretation of infant’s internal mental states—i.e., emotional, cognitive, and physiological states); (II); the overall attentional focus of the speech (i.e., infant focused, parent focused, or other experience focused) (III); and the affective state of speech (i.e., positive or negative maternal comments).

Examples of maternal comments and their ratings are presented in the Supplementary Material S1 (Methods).

### 2.4 Statistical analysis

Analyses were conducted using SPSS Statistics Version 27 for MacOs (IBM Ltd, UK). Prior to analyses, data were checked for missing data, outliers, accuracy, and normality. Main assumptions of normality and homoscedasticity were tested. If violated, data was either log-transformed or non-parametric analyses were conducted. Pearson’s chi-square (*χ*^2^) was used for categorical data, including sociodemographic and clinical variables, utilizing the *z*-test to compare the three groups. ANOVA was used to compare means for sociodemographic and clinical variables. Kruskal-Wallis test was used to compare means for the mother’s speech (as data were not normally distributed), with Bonferroni corrections for post-hoc comparisons between each of the groups. ANCOVA was used for covariate analyses of the interaction. Finally, hierarchical linear regressions were used for prediction modeling for maternal mentalization. Mean and standard error of the mean are presented in graphs.

## 3 Results

### 3.1 Sample characteristics

The characteristics of the specific sample presented in this study are consistent with previous descriptions of the broader PRAM-D cohort.^3,24,25^

#### 3.1.1 Antenatal risk factors

Antenatal characteristics are presented in Table 1. Women in the depression group, by definition, reported higher symptoms of depression on the BDI (*P* < 0.001); moreover, women both in the depression and history-only groups displayed more anxious symptoms on the STAI than healthy women (*P* < 0.001). However, only the depressed group showed a clinical level of anxiety (STAI-S score, 49.9 ± 13.7), and both control and history only groups displayed low or no clinical anxiety (STAI-S score, 26.1 ± 6.1 for control; 34.5 ± 11.7 for history-only) (cut off score 40^33,34^). A proportion in both the depression and history-only groups took antidepressants in pregnancy (respectively, 39.6% and 20%) before or after the baseline assessment. Most women in the depression and history-only groups had a history of recurrent depression prior to pregnancy, defined as two or more prior episodes (respectively, 55.3% and 62.5%, respectively).

Table 1 also presents sociodemographic/economics and clinical risk factors for antenatal and/or PND in the three groups. Women in both the depression and history-only groups had similar rates of exposure to childhood maltreatment, both of which were higher than healthy women (60.5% and 54.2%, vs. 12.8%, *P* < 0.001). However, only women in the depression group were also more likely to have lower education qualifications (vs. healthy women), be single (vs. history-only and healthy women), be unemployed (vs. healthy women), and to have a lower composite SES score (vs. healthy women) (*P* < 0.001−0.014; see Table 1). Due to the group differences in the history of childhood maltreatment and composite SES score (encompassing the above-listed sociodemographic and economic variables), these variables were included in subsequent univariate correlations and hierarchical regression models predicting maternal mentalization (see Tables 2 and 3).

#### 3.1.2 Postnatal outcomes

Postnatal characteristics are also presented in Table 1. There were no statistically significant group differences in mode of delivery, infant sex and age at the 8-week visit, feeding method or problems, or total duration of breastfeeding.

We evaluated PND using the SCID-I from birth through 8 weeks (*t*_1_), 8 weeks through 12 months (*t*_2_), and birth through 12 months (*t*_3_). Overall, women in both the depression and history-only groups were more likely to experience PND between birth and 12-months postnatally than women in the healthy group (*t*_3_: 42.9% and 36.4% vs. 7.5%, respectively: *P* = 0.001).

The NBAS orientation cluster at 6 days was significantly poorer in infants of women in both the depress and history-only groups than in infants of healthy women (6.3 ± 1.6 and 6.3 ± 1.8 vs. 7.6 ± 0.9, respectively; *F*(2, 113) = 13.12, *P* < 0.001; Tukey post hoc test *P* < 0.001 for both comparisons vs. healthy group).

Finally, and consistently with the results in an over-lapping sample,^3^ the dyadic synchrony evaluated with the CARE Index was significantly lower in the depression group compared with healthy women at 8 weeks (4.9 ± 3.3 vs. 5.7 ± 2.3) and in both the depression and history-only groups compared with healthy women at 12 months (45.6 ± 3.2 and 5.7 ± 2.8 vs. 7.9 ± 2.9) (*H*_(2)_ = 11.83, *P* = 0.003 at 8 weeks and *H*_(2)_ = 13.48, *P* = 0.001 at 12 months; *P* = 0.003 (healthy vs. depression at 8 weeks), *P* = 0.002 (healthy vs. depression at 12 months), *P* = 0.026 (healthy vs. history-only at 12 months), respectively).

As PND, the NBAS orientation cluster and dyadic synchrony variables differed between the groups, they were included in subsequent univariate correlations and hierarchical regression models predicting maternal mentalization (see Tables 2 and 3).

### 3.2 Only women depressed in pregnancy made significantly fewer mentalizing comments, but both groups of women with depression made significantly fewer comments focused on their infants’ experience

We compared scores on the PCAMs between depression, history-only, and healthy groups, at 8 weeks and 12 months postnatal (see Figure 1).

Women in the depression group made significantly fewer mentalizing comments (i.e. comments on their infant’s emotional, cognitive, and physiological states) compared with the healthy group both at 8 weeks (0.03 ± 0.01 vs. 0.07 ± 0.01, *P* = 0.002) and 12 months (0.02 ± 0.01 vs. 0.04 ± 0.01, *P* = 0.043) (*H*_(2)_ = 11.847, *P* = 0.003 at 8 weeks and *H*_(2)_ = 6.465, *P* = 0.039 at 12 months [see Figure 1A]).

Although no significant differences were found with regards to the history-only group at either timepoints, as Figure 1A shows they were numerically in the middle between the depression group and the healthy group, thus showing some evidence of difficulties in mentalizing.

With regard to the focus of maternal speech, both women in the depression and history-only groups made significantly fewer infant-focused comments compared with the healthy group at 8 weeks (0.53 ± 0.04 and 0.49 ± 0.05 vs. 0.67 ± 0.03, respectively; *H*_(2)_ = 12.647, *P* = 0.002; *P* = 0.015 (healthy vs. depression), *P* = 0.005 (healthy vs. history-only). However, at 12 months only depressed women made significantly fewer infantfocused comments compared with healthy group (0.49 ± 0.04 vs. 0.67 ± 0.04), with history-only groups again being numerically in the middle but not statistically different from either (*H*_(2)_ = 10.233, *P* = 0.006; *P* = 0.005 (healthy vs. depression), *P* = 0.132 (healthy vs. history-only), respectively).

Results on focus of the speech on their own experience or on other experience broadly follow this trend, and are presented in Supplementary Material S1 (Results).

### 3.3 Both groups of women with depression showed a decreased proportion of overall positive comments

As shown in Figure 1B, maternal depression and a history of depression were associated with a decreased proportion of positive comments compared with healthy women at 8 weeks (0.07 ± 0.01 and 0.08 ± 0.02 vs. 0.13 ± 0.01, respectively; *H*_(2)_ = 12.179, *P* = 0.002, *P* = 0.003 (healthy vs. depression), *P* = 0.043 (healthy vs. history-only)). However, at 12 months only depressed women made significantly fewer positive comments compared with the healthy counterpart (0.04 ± 0.01 vs. 0.7 ± 0.01; *H*_(2)_ = 6.637, *P* = 0.036, *P* = 0.032 (healthy vs. depression)). In contrast, no differences between groups were found in the proportion of overall negative comments at 8 weeks and 12 months (*H*_(2)_ = 0.003, *P* = 0.998, and *H*_(2)_ = 1.456, *P* = 0.483, respectively).

### 3.4 Variables associated with mentalization at 8 weeks and 12 months

Based on previous literature reporting the association between maternal mentalization and offspring developmental outcomes, we performed follow-up analyses on maternal mentalization.^16^ We therefore conducted univariate correlations between mentalization and identified risk variables that differed between groups (see Table 2). We then included all correlated variables in linear regression models (see Table 3).

As maternal BDI and STAI scores at baseline were highly correlated (*r* = 0.77; *P* < 0.001), we only included STAI score in the analyses because it correlated with mentalization at both timepoints. Thus, the final variables included in the hierarchical regression predicting maternal mentalization were: maternal SES score, maternal history of childhood maltreatment, STAI score during pregnancy, infant NBAS orientation score, dyadic synchrony at 8 weeks (for mentalization at 8 weeks) and 12 months (for mentalization at 12 months), and presence of MDD at 8 weeks (for mentalization at 12 months) (see Table 3).

At 8 weeks, SES cumulative risk score was significantly associated with mentalization and accounted for 5% of the variance; STAI score during pregnancy was also significantly associated with mentalization and increased the variance explained to 12%. Maternal history of maltreatment, NBAS orientation score, and dyadic synchrony at 8 weeks, each added approximately 3% to the variance explained, and together the model explained 20%; adding the maternal group (depression/history-only/healthy) add another 3% to the variance explained; however, these small effects did not significantly increase the prediction of mentalization in the model.

At 12 months, SES cumulative risk score was also significantly associated with mentalization and accounted for 5% of the variance; STAI scores during pregnancy and at 8 weeks each added 2−3% to the variance explained, while MDD at 8 weeks did not increase the variance explained; dyadic synchrony at 8 weeks and 12 months, each added 2−3% to the variance explained, and together the model explained 15%; adding the maternal group (depression/history-only/healthy) only added another 1%.

## 4 Discussion

This study investigates the impact of women’s clinical depression (either in pregnancy or in a lifetime before pregnancy) on cognitive biases (attentional focus and affective quality) and mentalizing features in the speech to their infants during mother-infant interaction at 8 weeks and 12 months postnatally.

We demonstrate that women who were depressed in pregnancy have decreased mentalizing ability at both 8 weeks and 12 months postnatally, and that both depression in pregnancy and a history of depression are associated with less speech focused on the infant’s experience and with decreased positive affect in the speech. In linear regression models, the reduced mentalization was explained mainly by maternal socioeconomic difficulties and clinical levels of anxiety in pregnancy, although childhood maltreatment, maternal anxiety at 8 weeks postnatally, reduced neonatal orientation skill, disrupted dyadic synchrony and PND all contributed to the variance explained. We show that mothers who experienced depression during pregnancy focus more on the self, are less able to mentalize and to focus on the infants’ experience and show less positive affection with their infants. Previous studies have shown that mothers with PND focus less on the infants’ experience, are less likely to comment on their infants’ mental state and to acknowledge their agency and intentions.^12,18^

Although our study found that women in both the depression and history-only groups are more likely to be postnatally depressed at 12 months than women in the healthy group (Table 1), which is consistent with the notion that antenatal depression and a history of depression are both risk factors for PND,^27^ the presence of PND itself is not associated with decreased mentalization at 12 months in the model, or in the univariate correlations when examined from birth through to 12 months, suggesting that women who experience PND alone may be protected in their interactions. As stated above, studies that have looked at women who experience PND have found less optimal communication with their infants via reduced mentalization and affective state in the speech,^12,17,18^ but the majority of these studies have not taken into account antenatal symptoms and/ or a history of depression. To date, other studies that have investigated the impact of perinatal depression on offspring outcomes have also found that antenatal depression is more predictive of outcomes, including offspring behavioral problems.^35,36^ The previous study with PRAM-D cohort and others that looked at both antenatal and postnatal symptoms in the context of mother−infant interaction found that antenatal depression is more predictive than PND in how the dyad will behave,^37,38^ possibly because antenatal depression may lead to disrupted fetal attachment and bonding early in the postpartum. Because antenatally depressed mothers often already experience stress, anxiety and negative feelings towards the fetus in pregnancy,^39^ and previous studies on PND did not measure depression in pregnancy, it is possible that impairments in mentalization and cognitive biases may be due to the continuation of this negativity postnatally rather than to specific postnatal effects. Of note, we had previously shown a reduced maternal sensitivity in this groups of women depressed in pregnancy,^3^ and mentalization is known to correlate with maternal sensitivity^40^; however, again our linear regression models suggest that this association is due to shared socioeconomic or clinical risk factors.

Women with a history of depression have mentalization skills that are intermediate between healthy and depressed, although not statistically significant from either, suggesting some subtler disruption in mentalization also in these women. Furthermore, they show decreased positive affection in their speech, fewer comments related to the infant’s experience and more comments not directly related to the current play context (i.e., removed in time and place), again indicating some disrupted mentalizing processes and cognitive biases in these women. Additionally, as previously reported, these women also show disrupted mother-infant interactions, with scores on the CARE-Index overlapping with those of mothers in the antenatal depression group.^3,18^ Taken together, evidence points towards difficulties in both behavioral and verbal features of the mother−infant relationship in the context of maternal antenatal depression and maternal history of depression.

As mentioned above, maternal socioeconomic difficulties and anxiety symptoms in pregnancy appeared to explain most of the variance in mentalization, and adding group (depression/history-only/healthy) to the model only added 1%−3% to the overall variance. These findings are in line with our previous study, which had already shown that SES score was the main variable predicting decreased quality of mother-infant interaction.^3^ More-over, another study analyzing cognitive biases in speech showed, that infant-directed negativity was not associated with infant’s behavior (e.g., fretfulness),^31^ and in our study the infants’ NBAS orientation score only just adds minimally to the mentalization variance explained at week 8, and it is not correlated with mentalization at 12 months. Since maternal antenatal anxiety has been associated with lower levels of maternal-fetal attachment,^41^ and with inadequate mother-infant emotion regulation, the results of our study confirm the important role of anxiety during pregnancy in maternal postnatal mental processes. Furthermore, studies have found that anxiety related to the developing attachment in pregnancy is associated with decreased mentalization.^42^ Overall, our results are in line with other studies that have shown that antenatal depression is commonly comorbid with clinical anxiety^43^; moreover, given that both antenatal depression and anxiety are shown to affect the quality of the postnatal relationship, it is likely that in our sample both sets of symptoms contributed to decreased mentalizing ability in the antenatally depressed mothers. One possible biological explanation of our findings, integrating the psychosocial processes discussed above, is that, during pregnancy mothers with a history of depression may be exposed to biological changes such as increased cortisol,^24^ and dysregulated oxytocin,^44^ an important hormone implicated in the formation of maternal behaviors that form the basis of the mother-infant bonding.^45,46^

There are a number of limitations that should be noted. First, since the speech scale was only used in one previous study,^31^ further validation is needed; however, the scale is based on well-described constructs,^12,13,47^ with good reliability between raters. Second, we have not evaluated mothers’ attachment to their own mothers, and many studies found an intergenerational pathway of parent attachment,^40^ suggesting a behavioral mirroring of their own parents.^48^ Third, although we evaluated whether there was an association between antidepressants and maternal mentalization, we were unable to assess the potentially differing effects of different classes of psychotropic medications such as anxiolytics or antipsychotics, as these were not used in our sample. Future studies with more heterogeneous medication use should investigate this further. More-over, no information was collected for the history-only group regarding the duration of depressive episodes and type of treatment received, and these could potentially have an effect on maternal communication postnatally. Finally, the sample size in our study was relatively small; even though we found statistically-significant differences in the speech features between groups, further replication would be beneficial.

In conclusion this study is the first to report on cogni-tive biases and mentalizing difficulties in the speech of mothers who are depressed in pregnancy or with a history of depression before pregnancy. Infants might be affected by a negative cognitive style of communication,^12^ and these patterns of communication may persist throughout their development. Moreover, mothers with impaired mentalization might be helped both in the antenatal and the postnatal period, through mentalization-based psychotherapies or social activity sessions that optimize the engagement with their infants, such as music and singing classes,^49,50^ especially since treating maternal depression alone is not sufficient for improving mother-infant relationship difficulties.^51^ Thus, we recommend both earlier detection of maternal depression in pregnancy and wider availability of these interventions in order to prevent the intergenerational transmission of vulnerability to psychopathology.

## Supplementary Material

Supplementary Material

## Figures and Tables

**Table 1:** Sample characteristics.

	Healthy group (*n* = 39−44)	History only group (*n* = 24−25)	Depression group (*n* = 41−46)	Statistical tests and significance	Post hoc differences
*Baseline (25 weeks gestation)*					
Age, years, mean (SD)	31.9 (4.5)	33.7 (5.8)	31.2(6.6)	F(2, 114) = 1.54, ***P*** = 0.220	—
Ethnicity^a^, white, *n (%)*	33 (77.3)	22 (88)	29 (63)	χ^2^(2) = 5.65, *P* = 0.059	—
White or white British, *n (%)*	33 (77.3)	22 (88)	29 (63)		—
Black or black British, *n (%)*	8 (15.2)	2 (8)	11 (24)		—
Mixed background, *n (%)*	3 (7.5)	1 (4)	5 (13)		—
Education, A level or higher, *n (%)*	41 (93.2)	21 (84.0)	32 (69.6)	**χ^2^(2) = 8.51, *P* = 0.014**	Healthy versus depression
Employment status, employed, *n (%)*	40 (90.9)	21 (84)	28 (60.9)	**χ^2^(2) = 12.39, *P* = 0.002**	Healthy versus depression
Marital status, married or cohabiting, *n (%)*	39 (88.6)	20 (80)	23 (50)	**χ^2^(2) = 17.59, *P* < 0.001**	Healthy versus depression, history-only versus depression
SES score, mean (SD)	0.43 (0.71)	0.28 (0.73)	−0.36 (1.13)	**H_(2)_ = 11.48, *P* = 0.003**	Healthy versus depression
Number of past episodes of MDD, >2, *n (%)*	—	15 (62.5)	26 (55.3)	χ^2^(1) = 0.34, *P* = 0.56	—
Antidepressant usage in pregnancy, yes, *n (%)*	—	5 (20)	19 (39.6)	χ^2^(1) = 2.86, *P* = 0.09	—
Parity, primiparous, *n (%)*	24 (54.5)	15 (60)	21 (45.7)	χ^2^(2) = 1.50, *P* = 0.473	—
BDI score at baseline, mean (SD)	3.4 (2.5)	5.3 (4.0)	19.1 (13.5)	***F(2,*106) = 37.67, *P* < 0.001**	Healthy versus depression, histoiy only versus depression
STAI-S score at baseline, mean (SD)	26.1 (6.1)	34.5 (11.7)	49.4 (13.7)	***F(2,*106) = 48.72, *P* < 0.001**	Healthy versus history-only, healthy versus depression, history-only versus depression
Maternal histoiy of maltreatment, yes, *n (%)*	5 (12.8)	13 (54.2)	26 (60.5)	**χ^2^(2) = 21.17, *P* < 0.001**	Healthy versus history-only, healthy versus depression
	**Healthy group *(n* = 23-41)**	**History only group *(n* = 17-24)**	**Depression group *(n* = 36-45)**	**Statistical tests and significance**	**Post hoc differences**
*Postnatal period*					
*6 days after delivery*					
Mode of delivery, vaginal, *n (%)*	32 (72.7)	19 (76)	36 (80)	χ^2^(2) = 0.65, *P* = 0.772	—
Infant sex, female, *n (%)*	22 (50)	10 (40)	21 (45.7)	χ^2^(2) = 0.65, *P* = 0.723	—
NBAS, orientation cluster at 6 days, mean (SD)	7.65 (0.9)	6.28 (1.78)	6.28 (1.59)	**F(2.113) = 13.12, *P* < 0.001**	Healthy versus history-only, healthy versus depression
*8 weeks after delivery*					
Infant age in days at 8-week assessment^a^, mean (SD)	68.16 (14.99)	81.18 (25.57)	79.93 (25.32)	H_(2)_ = 67.05, *P* = 0.029	—
Feeding method at 8 weeks, breast, *n (%)*	27 (61.4)	14 (58.3)	29 (41.3)	χ^2^(2) = 4.03, *P* = 0.134	—
Any feeding problems, yes, *n (%)*	9 (39.1)	9 (39.1)	13 (34.2)	χ^2^(2) = 0.22, *P* = 0.897	—
Antidepressant usage at 8 weeks, yes, *n (%)*	0 (0)	4 (16.7)	16 (34.8)	**χ^2^(2) = 18.48, *P* < 0.001**	Healthy versus history-only, healthy versus depression
SCID diagnosis of MDD between birth and 8 weeks, yes, *n (%)*	1 (2.3)	2 (8)	9 (19.6)	**χ^2^(2) = 7.40, *P* = 0.025**	Healthy versus depression
BDI score at 8 weeks, mean (SD)	3.07 (3.09)	5.08 (3.79)	11.63 (11.9)	***F(2,*107) = 13.31, *P* < 0.001**	Healthy versus depression, history-only versus depression
STAI-S score at 8 weeks, mean (SD)	25.36 (6.67)	34.04 (9.47)	42.1 (14.06)	***F*(2,106) = 25.5, *P* < 0.001**	Healthy versus history-only, healthy versus depression, history only versus depression
Dyadic synchrony score at 8 weeks, mean (SD)	5.73 (2.37)	4.43 (2.95)	4.19 (3.28)	**H_(2)_ = 11.83, *P* = 0.003**	Healthy versus depression
Infant difficulty score at 8 weeks, mean (SD)	3.82 (3.04)	4.22 (2.86)	3.64 (3.26)	H_(2)_ = 1.12, *P* = 0.571	—
*12 months after delivery*					
Infant age in days at 12-month assessment^a^, mean (SD)	396.17 (22.38)	421.41 (42.91)	405.91 (26.62)	**H_(2)_ = 7.57, *P* = 0.023**	Healthy versus history-only
Total months of breastfeeding at 12 months, mean (SD)	8.35 (4.1)	7.18 (3.86)	7.80 (3.85)	F(2, 90) = 0.54, *P* = 0.584	—
Antidepressant usage at 12 months, yes, *n (%)*	0 (0)	4 (17.4)	14 (32.6)	**χ^2^(2) = 15.27, *P* < 0.001**	Healthy versus history-only, healthy versus depression
SCID diagnosis of MDD between 8 weeks and 12 months, yes, *n (%)*	2 (5)	7 (31.8)	15 (35.7)	**χ^2^(2) = 12.10, *P* = 0.002**	Healthy versus history-only, healthy versus depression
Any SCID diagnosis of MDD between birth and 12 months, yes, *n (%)*	3 (7.5)	8 (36.4)	18 (42.9)	**χ^2^(2) = 13.73, *P* = 0.001**	Healthy versus history-only, healthy versus depression
BDI score at 12 months^a^, mean (SD)	3.054 (3.1)	6.73 (5.99)	11.08 (11.03)	***F(2,100)* = 10.64, *P* < 0.001**	Healthy versus depression
STAI-S score at 12 months, mean (SD)	26.62 (6.96)	34.45 (11.13)	40.40 (13.30)	***F(2,*100) = 16.15, *P* < 0.001**	Healthy versus history-only, healthy versus depression
Dyadic synchrony score at 12 months, mean (SD)	7.89 (2.87)	5.73 (2.84)	5.62 (3.21)	**H_(2)_ = 13.48, *P* = 0.001**	Healthy versus history-only, healthy versus depression
Infant difficulty score at 12 months, mean (SD)	2.84 (2.64)	2.45 (2.58)	2.71 (2.74)	H_(2)_ = 0.32, *P* = 0.853	—

***Note:*** Bold values indicate statistical significance. Abbreviations: BDI, Beck Depression Inventory; MDD, major depressive disorder; NBAS, Neonatal Behavioral Assessment Scale; SCID, Structured Clinical Interview for DSM-IV Disorders; SES, socioeconomic score; STAI-S, State−Trait Anxiety Inventory-State.

a In a previous study with the same sample but larger than this one, differences between groups were found for: ethnicity (history-only vs. depression), infant age at 8 weeks (healthy vs. depression and history-only vs. depression), infant age at 12 months (healthy vs. depression, history-only vs. depression) and BDI score at 12 months (healthy vs. depression, history-only vs. depression).

**Table 2:** Correlations between maternal and infant variables with group differences and maternal mentalizing.

	Mentalizing comments at 8 weeks	Mentalizing comments at 12 months
SES score^a^	0.26**	0.37**
History of maltreatment^b^	**−0.22** *	−0.20
BDI score during pregnancy^a^	**−0.39** **	−0.18
STAI-S score during pregnancy^a^	**−0.32** **	**−0.26** *
Antidepressants during pregnancy^b^	−0.12	−0.12
NBAS orientation score at 6 days^a^	0.25*	0.11
Postnatal MDD birth to 8 weeks^b^	0.02	**−0.26** *
BDI score at 8 weeks^a^	−0.13	−0.15
STAI-S score at 8 weeks^a^	−0.18	**−0.23** *
Antidepressants at 8 weeks^b^	−0.05	−0.04
Dyadic synchrony at 8 weeks^a^	0.35**	0.03
Postnatal MDD at 8 weeks^b^	−0.02	**−0.28** *
BDI score at 12 months^a^	**−0.24** *	−0.04
STAI-S score at 12 months^a^	−0.18	−0.15
Antidepressants at 12 months^b^	−0.15	0.03
Postnatal MDD 8 weeks to 12 months^b^	−0.04	−0.06
Dyadic synchrony at 12 months^a^	0.25*	0.26*

***Note*:** Bold values indicate statistical significance. Abbreviations: BDI, Beck Depression Inventory; MDD, major depressive disorder; NBAS, Neonatal Behavioral Assessment Scale; SES, socioeconomic score; STAI-S, State−Trait Anxiety Inventory-State.

* *P* < 0.05;

** *P* < 0.01.

a Spearman’s correlation coefficients are presented.

b Point-biserial correlation coefficients are presented.

**Table 3:** Hierarchical multiple regression explains variables associated with mentalization at 8 weeks and 12 months.

Maternal mentalization at 8 weeks (*n* = 85)				
Factors	Beta	95% CI	*R^2^*	*p*
SES score	0.013	0.001 to 0.026	0.05	**0.035**
SES score plus history of maltreatment	−0.018	−0.043 to 0.007	0.08	0.158
SES score plus history of maltreatment plus STAI score during pregnancy	−0.001	−0.002 to 0.000	0.14	**0.012**
SES score plus history of maltreatment plus STAI score during pregnancy plus orientation score	0.007	−0.002 to 0.016	0.17	0.123
SES score plus history of maltreatment plus STAI score during pregnancy plus orientation score plus dyadic synchrony at 8 weeks	0.003	0.000 to 0.007	0.20	0.082
SES score plus history of maltreatment plus STAI score during pregnancy plus orientation score plus dyadic synchrony at 8 weeks plus maternal groups:	0.047	−0.035 to 0.129	0.23	0.264
Healthy	Reference			
Depressed	−0.029	−0.064 to 0.006		
History only	−0.014	−0.044 to 0.016		
**Maternal mentalization at 12 months (n = 82)**				
**Factors**	**Beta**	**95% CI**	***R*** ^2^	***P***
SES score	0.013	0.000 to 0.025	0.05	**0.051**
SES score plus STAI score during pregnancy	−0.001	−0.002 to 0.000	0.07	0.172
SES score plus STAI score during pregnancy plus STAI score at 8 weeks	−0.001	−0.003 to 0.000	0.10	0.115
SES score plus STAI score during pregnancy plus STAI score at 8 weeks plus MDD at 8 weeks	−0.020	−0.100 to 0.059	0.10	0.614
SES score plus STAI score during pregnancy plus STAI score at 8 weeks plus MDD at 8 weeks plus dyadic synchrony at 8 weeks	−0.003	−0.007 to 0.002	0.12	0.278
SES score plus STAI score during pregnancy plus STAI score at 8 weeks plus MDD at 8 weeks plus dyadic synchrony at 8 weeks plus dyadic synchrony at 12 months	−0.004	−0.008 to 0.000	0.15	0.076
SES score plus STAI score during pregnancy plus STAI score at 8 weeks plus MDD at 8 weeks plus dyadic synchrony at 8 weeks and 12 months plus maternal groups:	0.124	0.067 to 0.181	0.16	0.642
Healthy	Reference			
Depressed	0.013	−0.028 to 0.054		
History only	−0.005	−0.040 to 0.030		

Abbreviations: MDD, Mayor depressive disorder; SES, socioeconomic score; STAI, State−Trait Anxiety Inventory.

## Data Availability

The data supporting this study’s findings will be available from the corresponding author, Lavinia Rebecchini, upon request after the completion of the study.

## Significant Outcomes

- Women depressed in pregnancy show fewer comments focused on their infant’s mental state at both 8 weeks and 12 months postnatally.
- Both women depressed in pregnancy and with a history of depression show less speech focused on the infant’s experience and decreased positive affect in their speech.
- Women’s socioeconomic difficulties and anxiety in pregnancy appears to be associated with decreased mentalization, while postnatal depression is not.

## Limitations

- We found statistically-significant differences in the speech features between groups, but the sample size is relatively small, and further replication would be beneficial.
- The speech scale is based on well-described constructs, with good reliability between raters, but it has only been used in one previous study, and we recommend further validation.
