# Supplementary material for: Women with depression in pregnancy or a history of depression have decreased quality of mentalization in the speech to their infants
Source: Acta Psychiatr Scand. Author manuscript; Available in PMC 2025 Dec 9. (PMC7618043; doi:10.1111/acps.13624)
Supplement: Supplementary Material [file EMS208242-supplement-Supplementary_Material_.docx]

Sample with video at 8 weeks and 12 months:

*n* = 131

Healthy group = 51

History-only group = 28

Depression group = 52

Video excluded:

*n* = 16 (12,2%)

Healthy group = 7

History-only group = 3

Depression group = 6

Baseline sample:

*n* = 115

Healthy group = 44

History-only group = 25

Depression group = 46

Attrition from baseline:

*n* = 1 (<1%)

Healthy group = 0

History-only group = 0

Depression group = 1

6-day assessment:

*n* = 114

Healthy group = 44

History-only group = 25

Depression group = 45

Attrition from baseline:

*n* = 14 (16%)

Healthy group = 1

History-only group = 3

Depression group = 10

8-week assessment:

*n* = 101

Healthy group = 43

History-only group = 22

Depression group = 36

Attrition from baseline:

*n* = 17 (19%)

Healthy group = 10

History-only group = 3

Depression group = 4

12-month assessment:

*n* = 98

Healthy group = 34

History-only group = 22

Depression group = 42

| **Supplementary Figure 1.** Psychiatry Research and Motherhood – Depression (PRAM-D) study participant flow chart. |
| --- |

**Supplementary study design:**

Inclusion criteria for the study: women of at least 18 years with a singleton pregnancy; for the depression group, a DSM-IV diagnosis of MDD in the current pregnancy, at or before 25 weeks’ gestation; for the history-only group, a history of MDD but no diagnosis of MDD throughout the entire pregnancy (if women developed depression in pregnancy after 25 weeks’, they were excluded from analyses); and, for healthy women, no current or past DSM-IV diagnoses. Exclusion criteria were as follows: uterine anomaly, obstetric complications, severe or relevant chronic medical conditions, history of psychosis or bipolar affective disorder and antidepressant usage at baseline (but not before or after baseline).

**Supplementary Methods:**

(I) Mentalizing comments included internal state agency comments such as: interpreting infant vocalizations, i.e., speaking on the infant's behalf (e.g., 'I am a happy baby'); sensitivity to mental states, i.e., cognitive state comments (e.g., 'you want that bottle'); feeling states, i.e., emotional state comments (e.g. 'you like this'); and somatic functioning, i.e., physiological state comments (e.g. 'you are hungry'). This dimension reflected the mother's overall ability to understand and attribute intent to the infant's internal states, vocalizations, emotions, cognitions, and behaviours. This category highlights a 'meeting of minds', the motivation, and skills apparent in parental discourse to share psychological and behavioural states with the infant and is in line with prior research on maternal mind-mindedness (Meins & Fernyhough, 1999).

(II) Overall attentional focus of the speech. The overall attentional focus of speech comprised three dimensions: (a) infant-focused speech; (b) parent-focused speech; (c) and other-focused speech.

a. Infant-focused speech included comments directed to, or about, the infant. External state agency comments were included in this category, which reflected the maternal capacity to identify the infant's attention and its focus (e.g., 'are you looking at the colours?'); motor movements (e.g., 'are you trying to stretch your arms out?'); and vocal expressions (e.g. repeating and response to vocalizations and mouthings: 'da da da' or 'is that a smile?'). Also included were comments on the infant's physical attributes, or general description (e.g., 'you have such beautiful eyes'; 'you have pink socks') and attention orienting and maintaining questions and comments (e.g., the use of greetings and songs).

b. Parent-focused speech predominantly captured specific attention-seeking comments directed to influence the infant's behaviour (based on a parental demand/goal) and differentiated based on infant's level of engagement. Also included in this category were comments made by the mother about herself, reflecting an explicit focus on her own thoughts, feelings and experiences (e.g., 'I'm not able to make you smile', 'Mummy's not as good as Daddy'). Finally, comments about the infant reflect an implicit focus on the mother's desire for control, in the form of strong commands and instructions directed towards correcting infant behaviour. Comments such as 'don't frown at me, smile like you smile at Daddy', suggest the infant perform in a way consistent with his/her mother's request. This perspective was taken from the previous work conducted by Murray and colleagues with mothers' speech (Murray, Kempton, Woolgar, & Hooper, 1993).

c. Other-focused speech included comments that were not directly related to the current play context and removed in time and place (e.g., 'I wonder what Daddy is doing', 'you had a long sleep'). Also coded in this category were: comments specific to the set-up and context of the interaction (e.g., presence of the camera), and those indicating implicit signs of discomfort/situation consciousness (e.g., 'how much longer?').

(III) Affective state of speech: utterances were coded for the degree to which statements were negative or positive, in order to establish the affective state across the 3-min speech sample.

**Supplementary Results:**

Differently from the overall mentalizing comments, women in both the depression and history-only groups made significantly fewer comments focused on their infants' experience compared with the healthy group at 8 weeks (0.53±0.04 and 0.49±0.05 vs. 0.67±0.03, respectively; *H_(2)_=*12.647, *P*=0.002; Bonferroni correction *P*=0.015 (healthy vs. depression), *P*=0.005 (healthy vs. history-only). However, at 12 months only depressed women made significantly fewer infant-focused comments compared with healthy group (0.49±0.04 vs. 0.67±0.04), with no difference between healthy and history-only groups *(H_(2)_*=10.233, *P*=0.006; Bonferroni correction *P*=0.005 (healthy vs. depression), *P*=0.132 (healthy vs. history-only), respectively). There was no difference between the depression and history-only groups both at 8 weeks and 12 months (all Bonferroni correction *P*=1.00).

The speech of depressed women was also significantly more focused on their own experiences compared with healthy women at 12 months (0.43±0.04 vs. 0.30±0.03; *H_(2)_*=7.41, *P*=0.025; Bonferroni correction *P*=0.023 (healthy vs. depression)), with no differences between healthy and history-only groups (0.3±0.04 vs. 0.4±0.04, Bonferroni correction *P*=1.00). There was no difference between groups at 8 weeks (*H_(2)_=*4.576, *P*=0.101). Only women in the history-only group made significantly more comments focused on other experiences (i.e., comments that were not directly related to the current play context and removed in time and place) while interacting with their infants, compared with healthy women at 8 weeks (0.18±0.03 vs. 0.10±0.02; *H_(2)_=*7.059, *P*=0.029; Bonferroni correction *P*=0.035 (healthy vs. history only)), with no difference between depression and healthy groups (0.14±0.002 vs. 0.1±0.02, Bonferoni correction *P*=0.24); also there were no differences between groups at 12 months postnatal (*H_(2)_*=3.935, *P*=0.14).

| **Figure 1c.** Mean scores across patterns of proportions of attentional focus of the speech at 8 weeks and 12 months postnatal. *P<0.05 **P<0.01. |
| --- |

*References*

Meins, E., & Fernyhough, C. (1999). Linguistic acquisitional style and mentalising development: The role of maternal mind-mindedness. *Cognitive Development*, *14*(3), 363–380.

Murray, L., Kempton, C., Woolgar, M., & Hooper, R. (1993). Depressed Mothers’ Speech to Their Infants and its Relation to Infant Gender and Cognitive Development. In *J. Child FiKhai Piyckat* (Vol. 34).
